# Developing and deploying climate-resilient maize varieties in the developing world

**DOI:** 10.1016/j.pbi.2018.05.004

**Published:** 2018-10

**Authors:** Jill E Cairns, BM Prasanna

**Affiliations:** 1 International Maize and Wheat Improvement Center (CIMMYT), Harare, Zimbabwe; 2 CIMMYT, ICRAF Campus, United Nations Avenue, Gigiri, Nairobi, Kenya

## Abstract

In sub-Saharan Africa (SSA) and Asia maize yields remain variable due to climate shocks. Over the past decade extensive progress has been made on the development and delivery of climate-resilient maize. In 2016 over 70 000 metric tonnes of drought-tolerant maize seed was commercialized in 13 countries in SSA, benefiting an estimated 53 million people. Significant progress is also being made with regard to the development and deployment of elite heat-tolerant maize varieties in South Asia. Increased genetic gain in grain yield under stress-prone environments, coupled with faster replacement of old/obsolete varieties, through intensive engagement with seed companies is essential to protect maize crops grown by smallholders from the changing climates in SSA and Asia.

## Highlights

• Climate-resilient maize has the potential to increase yield by 5–25% in Africa.
• Establishment of a large managed stress screening network facilitated gains in grain yield in stress-prone environments.
• Timelines for replacing old varieties with improved climate resilient varieties are reducing.
• Higher genetic gain and faster variety replacement required to increase yield and climate resilience.

## Graphical abstract

**Figure:**
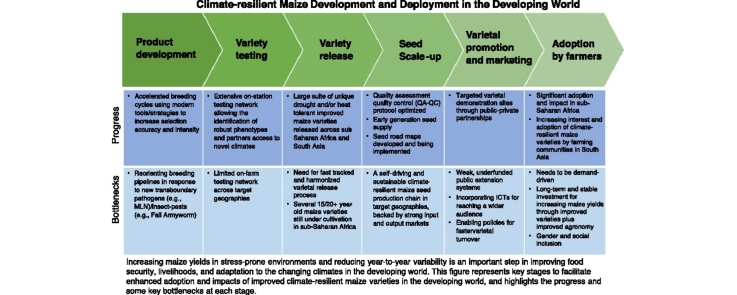

**Current Opinion in Plant Biology** 2018, **45**:226–230

This review comes from a themed issue on **AGRI 2017**

Edited by **David Edwards**

For a complete overview see the Issue and the Editorial

Available online 17th May 2018

**https://doi.org/10.1016/j.pbi.2018.05.004**

1369-5266/© 2018 The Authors. Published by Elsevier Ltd. This is an open access article under the CC BY license (http://creativecommons.org/licenses/by/4.0/).

## Introduction

Maize is the major source of food security and economic development in sub-Saharan Africa (SSA) and Latin America and the Caribbean (LatAm), and is among the top three crops in Asia. Over 300 million metric tonnes of maize is produced on over 90 million hectares across SSA, LatAm and Asia [1]. Average annual growth rate of the harvested maize area from 1993 to 2013 was 2.7% in Africa, 3.1% in Asia, and 4.6% in LA [1]. Even though the growth in area was accompanied by 2.4–5.6% increases in production, grain yields in these regions are still low with high year-to-year variability. In many regions of SSA and the Indo-Gangetic Plains, climate variability accounts for over 50% of the total variation in maize yields [2]. The predicted changes in temperature and precipitation will further accentuate the intensity and frequency of drought, increasing vulnerability of smallholder farmers to high risks associated with farming under rainfed conditions [3, 4]. Smallholder farmers continue to largely rely on open-pollinated varieties (OPVs) or outdated hybrids that were developed over 30 years ago [5^••^], limiting their ability to achieve food and nutritional security [6]. Climate-resilient maize has been specifically bred for increased tolerance to traits associated with a variable and changing climate, along with yield potential, defensive traits and consumer preferred traits [7^••^]. The main objective of this review is to present a brief update on the status and potential of climate-resilient maize in SSA and Asia, and identify key bottlenecks which need to be addressed to facilitate rapid development, scale-up and deployment.

## Drought and heat tolerant maize in SSA and Asia

The International Maize and Wheat Improvement Center (CIMMYT), in collaboration with national programs and the private sector, is intensively engaged in developing and deploying improved climate resilient maize varieties for tropical/subtropical environments in SSA, Asia and LatAm. New climate resilient maize in eastern and southern Africa (ESA) yield up to 20–25% more than current commercial varieties in on-farm trials under low-input and drought stress conditions [8]. During the severe El Niño induced-drought and heat stress in southern Africa in 2015–2016 crop season, climate-resilient maize yielded twofold more than key commercial hybrids in on-farm trials [9]. No yield penalty was observed in climatically good years. Crop modelling shows climate-resilient varieties will provide a yield advantage of 5–25% in many maize growing areas of ESA [4, 10].

Genetic gains achieved during the last few decades through conventional breeding have been, in part, associated with an expansion of phenotyping networks [11]. In ESA, selection for grain yield was previously conducted largely under optimal conditions, rather than under conditions representative of the target environments, while on-farm evaluations for proof of concept were limited [3]. Since 2009, the abiotic and biotic screening network was expanded to 59 locations across 11 countries. Phenotyping capacity for managed drought screening increased from 6 ha to 35 ha and low nitrogen stress screening from <10 ha to 47 ha [12, 13]. The large-scale, regional testing network allowed greater selection intensity for stress tolerance and maximized benefits of limited resources for maize important in ESA over a large area by allowing breeders access to managed stress facilities [12].

**Figure 1:**
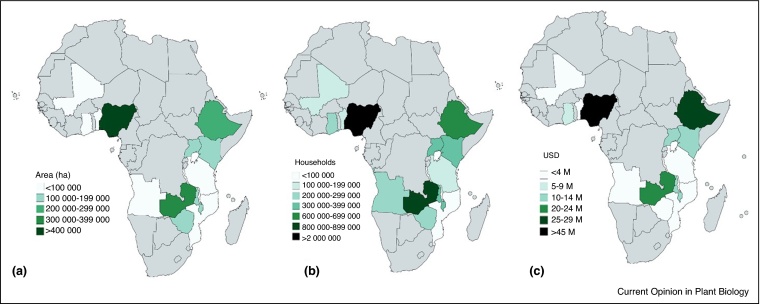
Estimated (a) maize area under climate-resilient maize, (b) number of households benefited from climate-resilient maize, and (c) economic value of increased maize production due to climate-resilient maize in 13 countries in sub-Saharan Africa.

Under the Drought Tolerant Maize for Africa (DTMA) project, over 230 climate-resilient maize varieties were released in 13 countries in SSA during 2007–2015. Of these, 63% were hybrids and 27% were improved OPVs. In 2016, over 70 000 tonnes of certified seed of climate-resilient maize varieties was produced in these countries, as compared to 30 768 tonnes in 2010 [4]. With the exception of Mozambique, Kenya and Zimbabwe where the production of climate-resilient maize seed has remained relatively constant between 2010 and 2016, adoption of climate-resilient maize has significantly increased over the years in the target countries in SSA. An estimated 85% of this seed (60 102 tonnes) was marketed in 2016, covering almost 2.5 million hectares (Figure 1a), and benefiting over 6 million households (or over 53 million people). This includes over 2 million households in Nigeria alone and 0.7 and 0.8 M households in Ethiopia and Zambia (Figure 1b).

The overall estimated economic value of increased maize production due to climate-resilient maize in Ethiopia was, at almost 30 M USD, over 10 M USD higher than previously estimated [14] (Figure 1c). A major factor associated with this success is the replacement of the old, climate-vulnerable maize varieties with improved climate-resilient hybrids, especially the hybrid BH661 [15^•^]. The high extension agent to farmer ratio (1:476) is likely to have further expedited the adoption, and associated economic benefits, of improved varieties [15^•^]. Recent post-ante studies in Ethiopia show the adoption of improved varieties is associated with increased per capita food consumption, and ultimately food security [16]. Similarly economic benefits were also higher than previously estimated in Zambia, Angola and Uganda. Small increases in yield in regions with a high frequency of drought can translate into a considerable increase in food security. For example, in two regions of Zimbabwe households that grew climate-resilient maize had more than nine months of food at no additional cost [17].

Most of the tropical maize growing areas in South Asia are highly vulnerable to drought and/or high temperature stress. Spring maize season, an important option for intensifying and diversifying cropping systems in South Asia, is particularly prone to severe heat stress during flowering/early grain filling stages [18]. Systematic efforts to develop elite Asia-adapted, heat tolerant maize cultivars were initiated in 2012 under the Heat Tolerant Maize for Asia (HTMA) project, implemented by CIMMYT in partnership with national maize programs in Bangladesh, India, Nepal and Pakistan, and 15 seed companies operating in Asia. A large heat-stress phenotyping network, comprising 23 sites in the four Asian countries, has been established. During 2015–2017, more than 50 elite heat stress tolerant, CIMMYT-derived maize hybrids have been licensed to public and private sector partners for varietal release, seed scale-up and deployment in the region.

## Ensuring greater gains in smallholder farmers’ fields

Recent estimates of genetic gain in grain yield within the ESA hybrid maize breeding pipeline of CIMMYT under experimental (on-station) conditions over a ten-year period were estimated at 109.4, 32.5, 22.7, 20.9 and 141.3 Mg ha^−1^ yr^−1^ under optimal conditions, managed drought, random drought, low N and MSV, respectively [12]. While these rates are equivalent to other regions of the world, yields remain lower [12]. With a constant linear increase at current yield gain trends, maize hybrid yields will only reach 3 Mg ha^−1^ in 17 years under random drought stress in experimental conditions.

**Figure 2:**
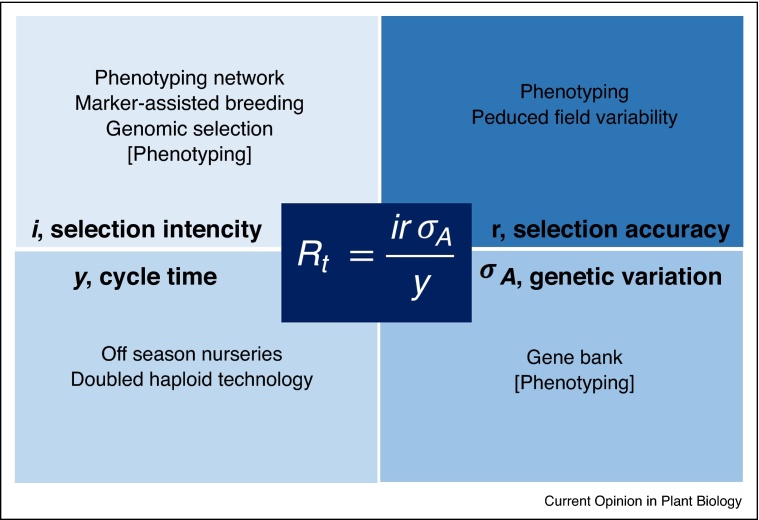
Technologies to increase genetic gain around the “breeders equation” [34], technologies in parentheses can indirectly contribute to the variable.

Increasing genetic gain under climate-related stresses will be essential to increase yields [19]. The ‘breeders equation’ provides the focus around which new technologies can contribute to increased genetic gain (Figure 2). One of the simplest ways to increase genetic gain is to reduce the breeding cycle time — if selection intensity, accuracy and variability remain constant, halving cycle time will double the genetic gain [20, 21••]. Breeding cycle times are 10 years or more, compared to less than five in temperate regions [5^••^]. Faster cycle times are also important for adaptation to emerging pests and diseases. Doubled haploid (DH) technology has now been optimized and deployed in SSA, reducing the time taken to develop parental lines [22]. More efficient systems for haploid induction and identification, coupled with chromosome doubling, are however essential to reduce costs of this process [23, 24].

A critical aspect to the design of breeding programs is the allocation of limited resources between population size and replication [25]. The development of low-cost, high throughput phenotyping tools have the potential to play an important role in reducing field costs, thus allowing resources to be allocated to generation and management of larger populations, enabling an increase in selection intensity within a fixed budget [26]. Recently there have been many advances in the development of high-throughput phenotyping tools for traits extensively used within breeding programs. Plant height sensors have been developed using a range of sensors including LiDAR, ultra-sonic sensors and RGB images [27, 28, 29]. Similarly image analysis has been used to quantify maize yield components [30] and diseases [31].

## Varietal replacement and adoption

For new climate-resilient varieties to contribute towards smallholders’ adaptation to climate variability, it is important to strengthen the seed systems. Delivering low-cost improved hybrids to smallholder farmers with limited purchasing capacity and market access requires that indigenous seed companies be supported with information on access to new products, besides adequate and reliable supplies of early-generation (breeder and foundation) seed of climate-resilient varieties [20].

A recent survey of product life cycles in SSA estimated average age is 14 years in East Africa, 15 years in Southern Africa and 16 years in West Africa [32]. When the time taken to develop varieties and for adoption is factored in, the process of variety development could have been initiated at least 25–30 years ago, based on the assumption of 8–10 years for variety development and 4–5 years for varietal registration and seed scale-up [20]. Each of the last three decades have been successively warmer than any preceding decade since 1850 [20], thus, these varieties were developed in a significantly different environment. However, there are some highly encouraging signs that the average age of varieties is decreasing in ESA [32]. Smale and Olwande [33] reported, based on a study in 2010, that the average weighted age of maize varieties in Kenya was 18 years, while Abate *et al.* [32], using a survey conducted in 2013, found the average weighted age was only 14 years. Appropriate government policies and adoption of progressive seed laws and regulations, are critical for improving smallholder farmers’ access to improved climate-resilient seed, and for overcoming key bottlenecks affecting the seed value chains, particularly in the area of policy, credit availability, seed production, germplasm and marketing.

## Conclusions

While further evidence is still required to document the risk-reduction benefits of the climate-resilient maize on the numbers of chronically poor farmers [7^••^], there is an increasing body of evidence confirming the benefits of climate-resilient maize to increase yields, reduce yield variability and, ultimately, increase food security. To increase genetic gains through maize breeding in the stress-prone tropics, and for enhancing the pace, precision and efficiency of breeding progress, judicious and effective integration of modern tools/strategies, especially high-density genotyping, high throughput and precision phenotyping, DH technology, molecular marker-assisted and genomic selection-based breeding, and knowledge-led decision-support systems, are vital. Emerging seed enterprises in SSA, Asia and LatAm also need to be strengthened to become more market-oriented and dynamic, to provide smallholders with greater access to affordable climate-resilient improved seed.

## References and recommended reading

Papers of particular interest, published within the period of review, have been highlighted as:

• of special interest
•• of outstanding interest
